# Dr. John Allen

**Published:** 1892-07

**Authors:** 


### DR. JOHN ALLEN.

AT the Twenty-fourth Annual Meeting of the Dental Society of the State of New York, held May 11 and 12, 1892, the following resolution was unanimously adopted:

WHEREAS, This Society has lost in the death of Dr. John Allen, of New York, one of its oldest and most worthy members, one who for nearly three-score years stood shoulder to shoulder with representative men in every laudable effort to promote the best interests of the dental profession, and who was recognized as the author of the highest art in one of our most important specialties; therefore,

*Resolved,* That this Society deeply mourns his loss and desires to extend to his family its truest sympathy.

(Signed) L. S. STRAW,
F. T. VAN WOERT,
O. E. HILL,
*Committee.*

